# Biweekly Hizentra® in Primary Immunodeficiency: a Multicenter, Observational Cohort Study (IBIS)

**DOI:** 10.1007/s10875-018-0528-5

**Published:** 2018-06-28

**Authors:** Alessandra Vultaggio, Chiara Azzari, Silvia Ricci, Baldassarre Martire, Valentina Palladino, Vera Gallo, Antonio Pecoraro, Claudio Pignata, Giuseppe Spadaro, Simona Graziani, Viviana Moschese, Antonino Trizzino, Giorgio Maria Boggia, Andrea Matucci

**Affiliations:** 1 Immunoallergology Unit, Department Medical-Geriatric, AOU Careggi, Brambilla, 3, 50134 Florence, Italy; 2 Department of Pediatric Immunology, Jeffrey Modell Center for Primary Immunodeficiency, University of Florence, Anna Meyer Children’s Hospital, Viale Pieraccini 24, 50139 Florence, Italy; 3 U.O.C. Oncologia e Ematologia Oncologica Pediatrica, Azienda Ospedaliero-Universitaria Policlinico, Bari, Italy; 4 Department of Paediatrics, AOU “Policlinico-Giovanni XXIII”, University of Bari “Aldo Moro”, Bari, Italy; 5 Department of Translational Medical Science, Pediatric Section, Federico II University, Naples, Italy; 6 Department of Translational Medical Sciences, Allergy and Clinical Immunology Center for Basic and Clinical Immunology Research (CISI), University of Naples Federico II, Naples, Italy; 7 Department of Pediatrics, Tor Vergata University, Policlinico Tor Vergata, Rome, Italy; 8 Department of Pediatric Hematology and Oncology, ARNAS Civico Di Cristina and Benfratelli Hospital, Palermo, Italy; 9 Medical Affairs, CSL Behring, Milan, Italy

**Keywords:** Primary immunodeficiency disease, 20% subcutaneous immunoglobulin, biweekly administration, IBIS study

## Abstract

Immunoglobulin G (IgG) replacement therapy is a standard treatment for patients with primary immunodeficiency diseases (PIDs). Hizentra®, a 20% human subcutaneous IgG (SCIG), is approved for biweekly administration for PIDs. The aim of the multicenter IBIS study was to prospectively investigate the efficacy of biweekly Hizentra® compared with previous IVIG or SCIG treatment regimens in patients with PIDs. The study consisted of a 12-month retrospective period followed by 12-month prospective observational period. The main endpoints included pre-infusion IgG concentrations, proportion of patients with serious bacterial infections (SBIs), other infections, hospitalizations due to PID-related illnesses, and days with antibiotics during the study periods. Of the 36 patients enrolled in the study, 35 patients continued the study (mean age 26.1 ± 14.4 years; 68.6% male). The mean pre-infusion IgG levels for prior immunoglobulin regimens during the retrospective period (7.84 ± 2.09 g/L) and the prospective period (8.55 ± 1.76 g/L) did not show any significant variations (*p* = 0.4964). The mean annual rate of SBIs/patient was 0.063 ± 0.246 for both prospective and retrospective periods. No hospitalizations related to PIDs were reported during the prospective period versus one in the retrospective period. All patients were either very (76.5%) or quite (23.5%) satisfied with biweekly Hizentra® at the end of the study. In conclusion, the IBIS study provided real-world evidence on the efficacy of biweekly Hizentra® in patients with PIDs, thus verifying the data generated by the pharmacometric modeling and simulation study in a normal clinical setting.

## Introduction

Primary immunodeficiency diseases (PIDs) are a heterogeneous group of chronic congenital disorders of the immune system where the immune system is compromised due to defects in one or more of its components [1]. The most common forms of PIDs with antibody deficiency (defined as primary antibody deficiency [PAD]) are common variable immunodeficiency (CVID), X-linked agammaglobulinemia (XLA), and autosomal recessive agammaglobulinemia (ARA) [2]. PIDs are estimated to affect 6 million people worldwide, although their exact prevalence remains unclear [3]. Patients with PIDs are more prone to recurrent, prolonged, and/or severe infections, and need timely diagnosis and treatment [4, 5].

Replacement therapy with intravenous human polyvalent IgG (IVIG) has been a gold standard treatment for patients with PIDs, in whom increasing serum IgG levels provide passive immunity to fight recurrent infections, thus significantly improving quality of life [6–8]. However, IVIG therapy is associated with a high incidence of adverse reactions, particularly in children, and it is often difficult to identify an appropriate vein for administration of therapy [9, 10]. Moreover, decreased efficacy with IVIG (the wear-off effect) towards the end of 3–4-week dosing cycle has also been quantitatively reported, which may be improved by increasing the IgG dose or reducing the dosing interval and/or a switch to subcutaneous immunoglobulin (SCIG) [11]. These challenges with IVIG therapy have led to the development of SCIG products, which are currently used in the treatment of patients with PIDs.

SCIG formulations infused weekly at lower IgG doses result in higher pre-infusion IgG values compared with monthly IVIG administration [12, 13]. Additionally, SCIG has improved patient compliance due to reduced adverse events (AEs), decreased time required for each individual infusion and ease of self-administration compared with IVIG [13–16]. Although, like most subcutaneous injections, SCIGs cause local reactions at the site of injection such as redness, itching, and swelling, these AEs tend to decline over time [17, 18].

Hizentra® (20% human SCIG, CSL Behring AG) is a SCIG formulation approved by the US Food and Drug Administration (FDA) and European Medical Agency for the treatment of patients with PIDs [19, 20]. A pharmacokinetic modeling and simulation study of Hizentra® in primary immunodeficiency predicted that biweekly administration of Hizentra® offers a viable alternative to weekly subcutaneous dosing, allowing more flexible and optimized IgG regimens in patients with PIDs [21]. The results of a retrospective record review of patients with PIDs treated with biweekly Hizentra® were consistent with the results of the pharmacokinetic modeling and simulation study [22]. However, a prospective clinical study to confirm these findings and to study the effect of a change in dosing regimen from weekly to biweekly on the frequency and seriousness of infections in normal clinical practice was warranted.

The Infusione Bimensile di Immunoglobuline Sottocute (IBIS; i.e., biweekly infusion of SCIG) study is the first prospective clinical study that aimed to compare the clinical and laboratory parameters of patients with PIDs receiving biweekly treatment with Hizentra® with their previous IgG-based treatments. The study also aimed to describe the profile of patients considered suitable for biweekly treatment with Hizentra®.

## Methods

### Study Design

**Fig. 1:**
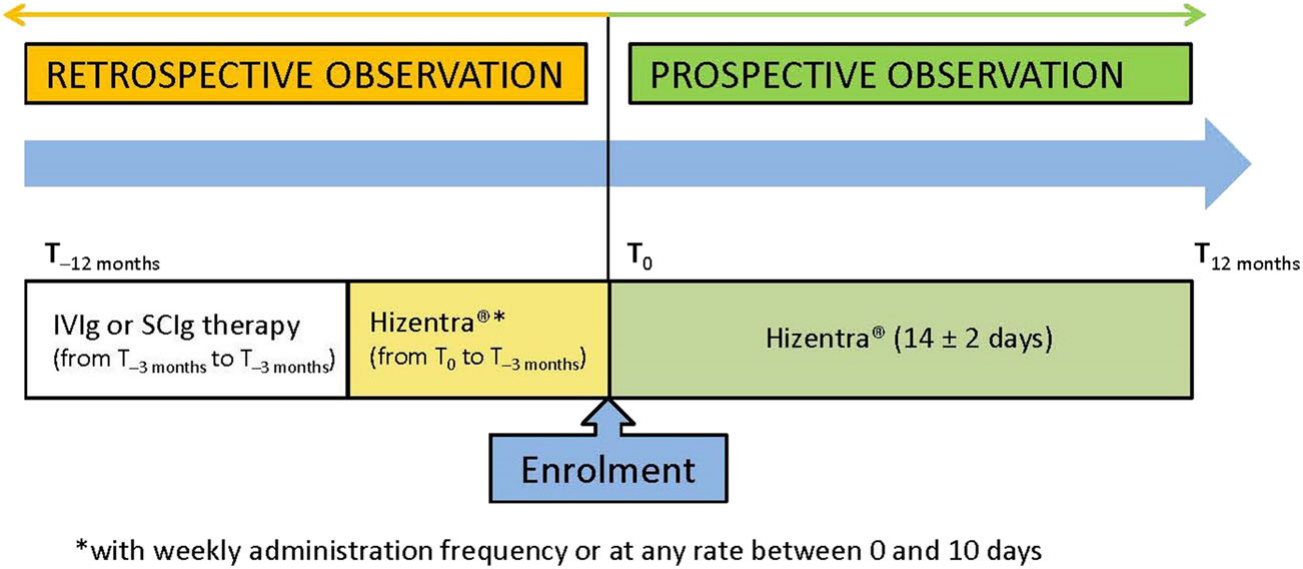
Study design. IVIG intravenous immunoglobulin, SCIG subcutaneous immunoglobulin, T time

This multicenter, prospective, observational cohort study with a retrospective analysis included patients with PIDs who underwent treatment with biweekly Hizentra®. Clinical data for each enrolled patient were collected for a 24-month observation period, which included 12 months each of retrospective and prospective observation (Fig. 1). The overall study duration was 18–6 months for enrolment of patients during which retrospective data were collected for up to 12 months prior to enrolment, followed by 12 months of prospective evaluation, which included data collection at 3, 6, and 12 months after enrolment using electronic case report forms. Enrolment in the study and treatment with Hizentra® were based on the judgment of the patients’ physician with the guarantee that the therapeutic choice was made in accordance with normal clinical practice.

Patients who met the following criteria were included in the study: patients (1–70 years of age) with hypogammaglobulinemia due to PIDs who required IgG replacement therapy at a dose that the investigator considered stable and protective against most infections, patients undergoing treatment with IgG (IVIG or SCIG) for at least 12 months and who had switched to Hizentra® at least 3 months before enrolment and who changed the frequency of administration of SCIG from weekly to biweekly (i.e., once every 2 weeks) upon enrolment, and patients for whom the minimum retrospective data were available for the 12 months preceding enrolment. Minimum retrospective data included at least one measurement of minimum plasma IgG concentration representative of the mean value during the period, data on previous IgG therapy (SCIG/IVIG, monthly doses, frequency of infusions, number of infusion sites for each session, infusion speed, number of pumps used (only for SCIG); number and type of serious bacterial (pneumonia, bacteremia/septicemia, osteomyelitis/septic arthritis, meningitis, visceral abscess) and other infections, and number of hospitalizations for PID-related illness. Exclusion criteria were any of the following: treatment with IVIG-type IgG therapy within 3 months prior to the enrolment visit; protein-losing illnesses; solid or onco-hematological neoplasms; concomitant treatment with high-dose systemic corticosteroids, immunosuppressive drugs, or plasma or other blood derivatives; positive viraemia for HIV-1, HIV-2, hepatitis C, or positive hepatitis B markers; pregnancy; and participation in clinical trials on investigational active ingredient or any other medicinal product that could interfere with the IgG replacement therapy. Concomitant or prior therapies for comorbidities related to PID or other pathologies were allowed during the study period.

The study was approved by the Comitato Etico Area Vasta Centro; Azienda Ospedaliera Universitaria Careggi, Firenze; and the Ethics Committees of each study center and was conducted in accordance with the Declaration of Helsinki and the current regulations for observational studies. All patients completed an informed consent form before participation in the study.

### Endpoints

The primary study endpoints included pre-infusion serum IgG concentrations for the retrospective and prospective periods; IgG concentration 7 days after subcutaneous injection of Hizentra® for the first three administrations during the prospective period; proportion of patients with serious bacterial (bacterial pneumonia, bacteremia/septicemia, osteomyelitis/septic arthritis, bacterial meningitis, visceral abscess, as defined by the US FDA [23]) and other infections and the number and types of infections, for both retrospective and prospective periods; and proportion of patients with hospitalizations due to illnesses related to PID, as well as the total number of hospitalizations during the retrospective and prospective periods. Secondary endpoints included determination of baseline characteristics of patients enrolled in the study (age, sex, weight, severity of PID) and manual ability of administration of Hizentra® during the retrospective period.

Clinical analyses of IgG levels were carried out in the laboratories of each center involved in the study; a central laboratory was not used for the prospective study testing.

Overall satisfaction with biweekly Hizentra® was evaluated by the patients’ physician during the follow-up visits. Patient satisfaction of biweekly Hizentra® treatment was assessed as follows: very satisfied, quite satisfied, little satisfied, and not satisfied.

### Statistical Analysis

The sample size for the study was determined based on feasibility criteria. Based on the number of patients with PIDs managed at the centers participating in this study, it was considered reasonable to include 30 patients (five patients/center on an average), and assuming 10% non-evaluable patients, it was expected to have 27 patients for the primary analyses. Precision of the estimates was evaluated in terms of confidence intervals (CIs) for mean IgG concentrations and the proportion of patients with infections based on reports of previous studies with Hizentra® [13, 14].

Statistical analyses for primary endpoints included evaluable patients for whom IgG plasma levels, infections, and hospitalizations for at least two of the three follow-up periods were available, while the secondary endpoints were evaluated in all enrolled patients who met the inclusion criteria. For primary objectives, the 95% CI limits were predicted for both observation periods. The 95% CI for the mean IgG concentration was expected to have a half-width of 0.5 g/L (expected mean ± standard deviation [SD] 8.10 ± 1.34 g/L), while the 95% CI for the proportions of patients with infections were expected to have a half-width of 18.5% (if the expected proportion was 60%) or 12.3% (if the expected proportion was 88%). Appropriate non-parametric statistical tests were used, wherever applicable, to determine the statistically significant changes (*p* = 0.05) between the two observation periods.

## Results

### Patient Disposition and Baseline Characteristics

A total of 36 patients were enrolled at seven centers in Italy, of which 35 patients (97.2%) continued the study. One patient withdrew informed consent the day after enrolment before starting the biweekly regimen, as they were no longer willing to participate in the study. All 35 patients had data available for analysis of IgG therapy prior to enrolment, infusion parameters during the biweekly Hizentra® administration and safety; 23 patients (63.9%) were considered evaluable for the primary analysis of IgG levels during the prospective period and 32 patients (88.9%) were evaluable for the primary analysis of infections/hospitalizations during the prospective period. Patients evaluable for primary analysis of IgG levels and infections/hospitalizations during the prospective period included all patients for whom data on pre-infusion IgG concentrations and infections/hospitalizations was available for at least two of the three follow-up visits at 3, 6, and 12 months after enrolment.

Of the 36 enrolled patients, 34 patients (94.4%) completed the study at the end of the prospective period, and two patients were terminated due to withdrawal of informed consent. Reasons for withdrawal of informed consent included no longer willing to participate in the study, and return to weekly regimen of Hizentra® after an AE which was not considered to be related to Hizentra®, reported by one patient each.

Mean ± SD age of the 35 patients who started the biweekly regimen of Hizentra® was 26.1 ± 14.4 years (age range 2–56 years; 25% of patients aged ≤ 14 years, 25% 14–24 years, and 50% ≥ 24 years), of which 24 were male (68.6%; Table 1). The mean ± SD disease duration at enrolment was 10.2 ± 8.9 years (range 1–40 years): 25% of patients reported PID for ≤ 4 years, 25% for 4–7 years, and 50% for ≥ 14 years (Table 1). CVID was the most prevalent type of PID (*n* = 20, 57.1%) followed by XLA (*n* = 9, 25.7%; Table 1).

**Table 1:** Baseline characteristics of patients included in the study

Characteristic	Evaluable patients, *n* = 35
Gender, *n* (%)	
Male	24 (68.6)
Female	11 (31.4)
Age at enrolment, years	26.1 ± 14.4
BMI, kg/m^2^	23.1 ± 4.7
PID type at enrolment, *n* (%)	
CVID	20 (57.1)
XLA	9 (25.7)
ARA	1 (2.9)
Other^a^	5 (14.3)
Disease duration at enrolment, years	10.2 ± 8.9
Comorbidities at enrolment, *n* (%)	
Autoimmune disease	3 (8.6)
Other PID-related pathologies	12 (34.3)

Values are presented as mean ± standard deviation unless otherwise stated

*ARA* autosomal recessive agammaglobulinemia, *BMI* body mass index, *CVID* common variable immunodeficiency, *PID* primary immunodeficiency, *SD* standard deviation, *XLA* X-linked agammaglobulinemia

^a^IgG subclass deficiency (*n* = 4) and DiGeorge syndrome (*n* = 1)

Fourteen patients reported at least one comorbidity related to PID or other relevant ongoing pathologies at enrolment, of which 8.6% of patients reported autoimmune disease and 34.3% reported other pathologies (the most frequent being chronic sinusitis [five cases] and bronchiectasis [three cases]; Table 1).

### IgG Therapy During the Retrospective Period

For the 36 patients enrolled in the study, the median duration of IgG therapy (IVIG and/or SCIG) was 6.0 years (interquartile range [IQR] 4.0–12.0). Twenty-three patients (65.7%) were self-administering IgG therapy, and 13 patients required assistance for administration of therapy from a relative.

The median duration of weekly treatment with Hizentra® was 2.4 years (IQR 1.2–3.2); 77.1% of patients (*n* = 27) were administered Hizentra® every 7 days, 17.1% (*n* = 6) every 10 days, and 5.7% (*n* = 2) every 6 days. At enrolment, 20% of patients (*n* = 7) had received IVIG therapy every 3–4 weeks, the median exposure duration of which was 5.7 years (IQR 0.1–17.5).

The main reported reasons for switching from weekly in the retrospective period to a biweekly Hizentra® regimen during the study were better compatibility with personal needs (*n* = 22, 62.9%), easily controllable disease (*n* = 13, 37.1%), low body weight of patients (*n* = 9, 25.7%), and pediatric patients (*n* = 8, 22.7%).

### Pre-Infusion IgG Concentrations

A total of 23 patients were evaluable for pre-infusion IgG concentrations at the end of the study. The median (IQR) pre-infusion serum IgG concentration during the retrospective period was 8.03 (7.10–9.25) g/L (*n* = 23). Median (IQR) pre-infusion IgG concentrations during the prospective observation period at 3, 6, and 12 months after administration of biweekly Hizentra® were 8.57 (8.05–9.79) g/L (*n* = 17), 8.15 (7.64–9.45) g/L (*n* = 21), and 7.98 (7.07–9.69) g/L (*n* = 21), respectively (Table 2).

**Table 2:** Pre-infusion IgG concentrations during biweekly Hizentra® dosing regimen

Pre-infusion IgG concentration (g/L)	*n*	Mean ± SD	Median (IQR)	95% CI
12-month retrospective period	23	7.84 ± 2.09	8.03 (7.10–9.25)	6.94, 8.74
3-month follow-up	17	9.25 ± 1.99	8.57 (8.05–9.79)	8.23, 10.28
6-month follow-up	21	8.65 ± 1.94	8.15 (7.64–9.45)	7.77, 9.53
12-month follow-up	21	8.57 ± 1.99	7.98 (7.07–9.69)	7.66, 9.48
12-month prospective period	23	8.55 ± 1.76	7.94 (7.36–9.67)	7.79, 9.31
Intra-patient variation between prospective and retrospective periods	23	0.71 ± 2.81	0.10 (− 0.82–1.22)	− 0.51, 1.92

*CI* confidence interval, *IQR* interquartile range, *SD* standard deviation

Median (IQR) intra-patient variation in pre-infusion IgG concentrations between the retrospective and prospective IgG values was 0.10 (− 0.82 to 1.22) g/L. No significant variations in mean ± SD pre-infusion IgG levels between prior regimens (7.84 ± 2.09) and biweekly Hizentra® (8.55 ± 1.76) were found (*p* = 0.4964, signed rank test).

During the retrospective period, treatment with Hizentra® was administered with a median (IQR) dose per infusion of 6 (4–8; mean ± SD 5.9 ± 3.1 g) g, median duration of each infusion was 1 (0.8–1.7) h, and median infusion speed for each session was 20 (12.5–22.5) mL/h. Two infusion sites were used during each session for 33 patients (94.3%), whereas a unique infusion site was used for 2 patients (5.7%).

The median (IQR) duration of exposure to biweekly Hizentra® was 12 (11.8–12.5) months, median dose per infusion session was 10 (8–12; mean ± SD 9.8 ± 3.6 g) g, median duration of each infusion was 1.6 (1.3–2.0) h, and median infusion speed for each session was 25 (18–36) mL/h. Two infusion sites were used during each session for 25 patients (71.4%), three infusion sites were used for 6 patients (17.1%), and four infusion sites were used for 3 patients (8.6%); only 1 patient (2.9%) used a single infusion site.

The slight reduction in the monthly dose of Hizentra® observed during the prospective phase (median dose of 10 g/infusion every 2 weeks) compared with the retrospective period (median dose of 6 g/infusion every week) was due to simplicity in the administration of the drug; in many cases, a 10-g (50 mL) vial of Hizentra® was used every 2 weeks.

### Bacterial and Other Infections

Among the 32 patients evaluable for infections (patients with available data on infections for at least two follow-up visits who did not interrupt biweekly therapy with Hizentra®), two patients (6.3%) reported a serious bacterial infection (SBI; visceral abscess and bacterial pneumonia) during the prospective period, and bacterial pneumonia was reported by two patients (6.3%) during the retrospective period. The mean (± SD) annual rate of SBIs/patient during the prospective and retrospective periods was identical (0.063 ± 0.246) and was calculated taking into account an episode of pneumonia which occurred in the prospective period but was erroneously reported elsewhere in the database and was assessed as an SBI after database lock.

At least one non-serious bacterial and non-bacterial infection (other infections) was reported in 24 patients (75%; 95% CI 56.60, 88.54) each during the retrospective and prospective observation period, and the total number of other infections reported during the prospective and retrospective period were 62 and 40, respectively (Table 3). The most prevalent infections during the prospective study period were pharyngitis (14 cases), sinusitis (10 cases), and bronchitis (8 cases), and during the retrospective period, these were pharyngitis (8 cases) and bronchitis (12 cases).

**Table 3:** Other infections (bacterial nail disorder, bronchitis, cutaneous papule, diarrhea and gastroenteritis, ear pain, epididymitis, flu and cough, genital candidiasis, herpes labialis, influenza, lower rim lesion, oral aphthae, oxyuriasis, periocular herpes, scarlet fever, streptococcus infection, tracheitis, urinary tract infections, vulvovaginitis) reported in patients during the prospective and retrospective study periods

Other infections	Retrospective period, number of cases (%)	Prospective period, number of cases (%)
Total number of other infections	40 (100)	62 (100)
Bronchitis	12 (30.0)	8 (12.7)
Rhinitis	4 (10.0)	5 (7.9)
Pharyngitis	8 (20.0)	14 (22.2)
Laryngitis	0 (0.0)	3 (4.8)
Otitis	1 (2.5)	1 (1.6)
Sinusitis	4 (10.0)	10 (15.9)
Conjunctivitis	1 (2.5)	0 (0.0)
Other type of infection	10 (25.0)	21 (33.9)

The median (IQR) annual rate of other infections during the retrospective and prospective observation period was 1.000 (0.50–2.00) and 1.767 (0.448–2.974). Median increase in the annual rate of other infections during the prospective period was not statistically significant (0.047; *p* = 0.0632, signed rank test).

Antibiotic therapy for bacterial infections (serious and non-serious) was reported by 60% of patients (*n* = 21) during the retrospective period and 62.9% (*n* = 22) during the prospective period. Median (IQR) duration of antibiotic therapy for serious and non-serious infections was 6 (0–20) days (*n* = 35) during the retrospective period, and 7 (0–16) days during the prospective period (*n* = 34).

### Number of Hospitalizations

No hospitalizations related to PID were reported during the prospective study period, and one patient reported hospitalization due to PID-related pathologies (pneumonia) during the retrospective period.

### Treatment Satisfaction

At the 12-month follow-up visit, 76.5% of patients (*n* = 26) were very satisfied and 23.5% (*n* = 8) of patients were quite satisfied with biweekly Hizentra®. Only one patient, who switched back to weekly administration of Hizentra® 22 days after enrolment, remained unsatisfied, as reported at the 6-monthly follow-up visit. Treatment with biweekly Hizentra® was completed by 94% of patients (*n* = 33), while 5.7% of patients (*n* = 2) switched back to the previous weekly regimen (after 22 and 261 days, respectively). Reasons for the switch, as reported by the treating physicians, were major discomfort at the injection site (*n* = 1) and mottled cutaneous thoracoabdominal vein reticulum (*n* = 1; an AE not considered to be related to Hizentra®).

The patient who experienced major discomfort at the injection site was being treated with 12 g Hizentra® every 14 days, administered using two pumps in parallel for three infusion sites, with a cumulative rate of 32 mL/h. After 22 days with biweekly Hizentra® treatment, therapy was switched to 6 g Hizentra® every 7 days (no information is available on the infusion parameters). The pain resolved when the patient was switched back to weekly Hizentra® treatment.

The patient with mottled cutaneous thoracoabdominal vein reticulum was being treated with 8 g Hizentra® every 14 days, administered using one pump for two infusion sites, with a cumulative rate of 40 mL/h. After 261 days with biweekly Hizentra® treatment, therapy was switched to 4 g Hizentra® every 7 days, administered using one pump for two infusion sites, with a cumulative rate of 40 mL/h. The mottled cutaneous thoracoabdominal vein reticulum disappeared on return to weekly Hizentra® administration.

### Safety Evaluation

During the prospective period, 22.9% of patients (*n* = 8) reported 11 AEs, of which adverse drug reactions (ADRs) were reported by two patients (edema at injection site) and serious adverse events (SAEs) were reported in one patient (pneumonia). The edema reported at the injection site by the two patients was mild and expected.

## Discussion

This is the first prospective, multicenter, observational study providing real-world evidence of the efficacy and safety of biweekly Hizentra® in patients with PIDs. The results of this study showed that biweekly administration of Hizentra® maintained stable IgG concentrations in patients with PIDs that were comparable with weekly SCIG administration. A study investigating the pharmacokinetics and safety of biweekly 16% SCIG in patients with PAD reported that the treatment was well tolerated with stable serum IgG levels [24]. Other pharmacokinetic and retrospective studies have reported that biweekly 20% SCIG therapy is well tolerated and results in stable serum IgG levels [22, 25]. The results of the present study are consistent with these observations and further show that despite a slight reduction in the monthly dose of SCIG observed during the prospective phase (median dose/infusion/2 weeks: 10 g) compared with the retrospective period with weekly administration (median dose/infusion/week: 6 g), IgG serum concentrations remained constant.

Regular monitoring of the patient’s serum IgG levels after administration of a new therapy can help in measuring the response to therapy. SCIG therapy is generally initiated at a dose of 100–150 mg/kg/week, and the dose is subsequently adjusted based on the serum IgG trough levels achieved for individual patients [26–28]. It is recommended that during treatment with IgG, the dose of IgG should be titrated to achieve a serum trough level of about 6–8 g/L in patients with PIDs to provide protection against infections [29, 30]. Pre-infusion serum IgG levels were monitored at regular intervals during the present study and showed that biweekly administration of Hizentra® maintained the pre-infusion trough levels of IgG above 7.5 g/L throughout the prospective study period.

During the present study, no relevant changes in terms of infections and hospitalizations were observed between the two groups, and there was no increase in the number of days of antibiotic therapy for infections in the prospective period compared with the retrospective phase of the study. However, it should be noted that the annual rate of infections (as well as any other AEs) calculated for the retrospective period may have been underestimated, as the data were collected retrospectively (recall bias). Also, other clinical findings erroneously reported as infections during the study may have been related to inflammatory comorbidities frequently occurring in patients with PIDs.

The mean annual rate of SBIs/patient during the prospective and retrospective period of the present study was identical and comparable to previously published studies with Hizentra® [13]. Moreover, rate of SBIs during the prospective study period was well below the threshold (< 1 SBI) recommended by the US FDA to show efficacy of IgG therapy [23]. Also, the percentage of patients with at least one other infection reported in the two phases of the present study was comparable with that reported in the phase III study (75 versus 78.3%) [13]. The choice of both retrospective and prospective observation windows, accounting for 12 months each, was intended to limit potential biases linked to the seasonal nature of the incidence of infections.

Treatment with biweekly Hizentra® progressively showed a high degree of patient satisfaction during this study. At the 12-month follow-up visit, all patients were at least quite satisfied, with the majority of patients being very satisfied (76.5%) with the biweekly regimen, and 94% of patients chose to continue the twice-monthly treatment protocol. Only one patient preferred to switch back to the weekly Hizentra® regimen because of discomfort experienced during the biweekly administration due to increased volume of administration. It has been documented that patients with PIDs may prefer a regimen with more frequent infusions at a lower volume [31]. With several options available for the administration of IgG with different dosing frequencies, patients can select the most suitable method based on individual preference [32]. Even though the study was not designed to monitor patient quality of life, overall, these results suggest favorable effects of the biweekly Hizentra® regimen.

In the present study, biweekly Hizentra® was well tolerated in patients with PID. Two patients experienced edema at the injection site (ADR) and one patient reported pneumonia (SAE). However, these events did not interfere with the study progression. Furthermore, it was observed that the SAE (pneumonia) reported in one patient was not related to the study medication, but was due to the primary immunodeficiency itself. These AEs reported with SCIG administration are less extensive than those caused by intravenous infusion [14]. Edema at the injection site is an expected event considering the high volume of solutions injected subcutaneously, which can be mitigated by dividing the dose to be administered among several infusion sites [19].

The main limitation of the present study was the possible introduction of bias for data collected retrospectively, especially due to missing or incomplete data. This was controlled for by strictly allowing only patients with a minimum required set of retrospective data available to be included in the study. Also, the IBIS study did not determine patients’ quality of life.

In conclusion, the IBIS study highlights the benefits of a biweekly Hizentra® treatment regimen in patients with PIDs in a clinical setting. The study showed that switching from weekly administration (or at any rate with a frequency of every 0–10 days) of Hizentra® to once every 2 weeks (biweekly) neither compromised serum IgG levels nor the rate of infections and hospitalizations.
